# Accounting for Tube Hematocrit in Modeling of Blood Flow in Cerebral Capillary Networks

**DOI:** 10.1155/2019/4235937

**Published:** 2019-08-18

**Authors:** Nikolai D. Botkin, Andrey E. Kovtanyuk, Varvara L. Turova, Irina N. Sidorenko, Renée Lampe

**Affiliations:** ^1^Fakultät für Mathematik, Technische Universität München, Boltzmannstr. 3, 85748 München, Germany; ^2^Klinikum Rechts der Isar, Technische Universität München, Ismaningerstr. 22, 81675 München, Germany; ^3^Far Eastern Federal University, Sukhanova st. 8, 690950 Vladivostok, Russia

## Abstract

The aim of this paper consists in the derivation of an analytic formula for the hydraulic resistance of capillaries, taking into account the tube hematocrit level. The consistency of the derived formula is verified using Finite Element simulations. Such an effective formula allows for assigning resistances, depending on the hematocrit level, to the edges of networks modeling biological capillary systems, which extends our earlier models of blood flow through large capillary networks. Numerical simulations conducted for large capillary networks with random topologies demonstrate the importance of accounting for the hematocrit level for obtaining consistent results.

## 1. Introduction

Simulation of blood circulation in large capillary networks is a challenging task. Realistic modeling of microvessel structures should take into account not only sophisticated topologies of blood vessel networks but also correct hydraulic resistance of microvessels. The latter is characterized by the apparent blood viscosity which depends on the vessel diameter as well as the discharge and tube hematocrit. The discharge hematocrit is the volume fraction of the red blood cells (RBCs) in the blood delivered by the flow in the vessel. The tube hematocrit is the volume fraction of RBCs that are inside the vessel at a given time instant. The discharge hematocrit is larger than the tube one because the velocity profile in the radial direction is nonuniform; namely, the RBCs velocity is higher than the mean bulk flow speed, which is called the Fåhraeus effect [1, 2]. The velocity profile in the radial direction is affected by the presence of the endothelial surface layer (ESL) [1].

The importance of accounting for the hematocrit level in blood flow simulations attracts attention of many researches. Animal models are utilized, for example, to measure and analyze the distributions of cell velocity and cell flux in the capillary network for different values of systemic hematocrit [3]. By using fluorescent microscopic analysis of rat cerebral capillary networks, the influence of hematocrit on mean RBC capillary velocity and mean arterial pressure can be assessed [4]. Moreover, the effect of hematocrit can be investigated in artificial microvascular branching networks [5]. A combination of an animal model with an effective iterative algorithm allows for finding the distribution of discharge hematocrit and blood flow velocity in a cerebrocortical microvascular network [6]. This approach takes into account the heterogeneity of blood flow and partitioning of red cells at bifurcations.

The current paper is related to modeling of computer-generated blood microvessel networks with vessel diameters less than 10 *μ*m. This is motivated by our previous work on simulating cerebral blood flow of preterm infants [7]. In such thin blood vessels, RBCs move in single file. Due to their ability to deform, RBCs can pass through vessels down to 2.7 *μ*m in size, which is less than their diameter, without damaging their membrane [8]. The hematocrit level and the shape of single erythrocytes during their motion in a capillary with diameter less than 8 *μ*m depend on RBC velocity [9].

Two approaches to mathematical modeling of RBC transport through microvessels can be mentioned.

The first one is based on continuum models [7, 10], where erythrocytes are considered as a homogeneous substance, and the RBC motion is described as a two-phase blood flow; namely, the erythrocyte homogeneous substance moves in the central core of the vessel, whereas the plasma fraction moves in a cell-free layer formation near the wall. Reasonable assumptions allow for deriving an explicit formula for the hydraulic resistance of a single capillary [7], which is very important for the simulation of blood flow in large capillary networks. A model derived in [10] studies the relations between the tube hematocrit level, vessel diameter, and apparent viscosity.

The second approach relies on discrete models [11, 12], where the effect of each erythrocyte is taken into account. The capillary blood flow is considered as single-file flow of red cells in blood plasma playing the role of lubrication and filling gaps between the erythrocytes. Such an ansatz [11] is used to develop a model resulting in an efficient algorithm for computing the pressure and flow field as well as the hematocrit distribution in simplified capillary networks. This approach shows a strong influence of single-file arrangement of RBCs on flow behavior. A coupled model describing the delivery of oxygen to tissue cells is considered [12] at the scale allowing to take into account the size and shape of individual RBCs as well as their deformation. The proposed approach [11, 12] takes into account the level of hematocrit in simulations of blood flow.

In the present paper, the approaches described in [7, 11] are combined to obtain an analytical formula for the computation of blood flow resistance in microvessels. This formula accounts for gaps between RBCs and, therefore, reflects the dependence on tube hematocrit. The tuning and validation of this formula are performed using hydrodynamical computations based on the representation of the cell-plasma mixture as a fluid with two different viscosities (much larger viscosity for blood cells). A very good consistency of the analytically computed values with the numerical results is obtained. An example of finding the pressure distribution in a relatively large capillary network (the germinal matrix or the whole brain), accounting for the level of tube hematocrit, is presented. The ability to account for the hematocrit level significantly enhances the algorithm proposed in [7] for finding the pressure distribution in the germinal matrix. Numerical experiments show a significant influence of the hematocrit level on the pressure distribution.

## 2. Continuous Model of Red Cell Transport

**Figure 1:**
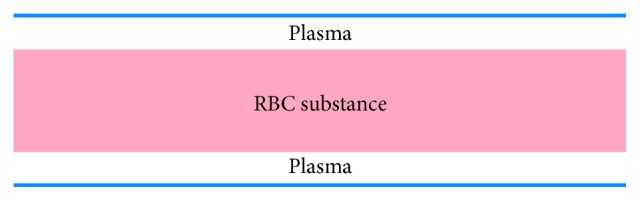
Schematic representation of continuum stream of erythrocytes in a capillary (the axial section is shown). The gaps between erythrocytes are ignored. The motion occurs due to the lubrication plasma layer between erythrocytes and vessel wall.

In [7], the transport of red cells in capillaries was modeled as a continuum flow with spatially variable viscosity. A high viscosity was assigned to the central part of the capillary, RBC substance, whereas the layer between the RBC substance and capillary wall was assigned with a small viscosity typical for blood plasma (Figure 1.)

Such an approach is motivated by the results of [13] claiming that a rigid body moving in a fluid can be replaced with another fluid whose viscosity tends to infinity. Ignoring gaps between the red cells was explained by small length of gaps compared to a high velocity of RBCs. Thus, the resistance of a capillary was assumed to be independent on the hematocrit in a first approximation.

The viscosity as function of the vessel's radius was chosen as follows:

(1) $$\begin{array}{c} \mu \left(r\right)=\left\{\begin{array}{cc} \mu_{1}, & \mathrm{if}\;r_{0}\leq r\leq r_{\text{c}},\\ \mu_{2}, & \mathrm{if}\;r<r_{0}, \end{array}\right. \end{array}$$

where *r*_c_ is the radius of the capillary, *r*_0_ is the radius of the RBC substance, and *μ*_1_ and *μ*_2_ are the viscosities of blood plasma and the RBC substance, respectively. Typically, *μ*_1_=0.001 Pa · s and *μ*_2_=0.1 Pa · s. The last high value is used here to make the RBC column effectively rigid. It should be noted that further increasing *μ*_2_ has practically no effect.

The velocity profile corresponding to (1) was derived in [7, 14], under some assumptions, as follows:

(2) $$\begin{array}{c} v\left(r\right)=\left\{\begin{array}{cc} \frac{A\left(r^{2}-r_{\text{c}}^{2}\right)}{4\mu_{1}}, & \mathrm{if}\;r\geq r_{0},\\ \frac{A\left(r_{0}^{2}-r_{\text{c}}^{2}\right)}{4\mu_{1}}+\frac{A\left(r^{2}-r_{0}^{2}\right)}{4\mu_{2}}, & \mathrm{if}\;r<r_{0}, \end{array}\right. \end{array}$$

where *A*=Δ*p*/*L* and Δ*p* is the pressure drop at the capillary of the length *L*. The total flux is given by the following formula:

(3) $$\begin{array}{c} q=-\frac{\pi \Delta p}{8L}\left(\frac{r_{\text{c}}^{4}-r_{0}^{4}}{\mu_{1}}+\frac{r_{0}^{4}}{\mu_{2}}\right), \end{array}$$

and the resistance reads as follows:

(4) $$\begin{array}{c} \hat{R}=\frac{8L}{\pi }{\left(\frac{r_{\text{c}}^{4}-r_{0}^{4}}{\mu_{1}}+\frac{r_{0}^{4}}{\mu_{2}}\right)}^{-1}. \end{array}$$

In [7] (see the end of Section 2 and references there), the following relation between *r*_0_ and *r*_c_ was proposed:

(5) $$\begin{array}{c} r_{0}=0.3\;\mu \text{m}+0.8r_{\text{c}}, \end{array}$$

and arguments for the reliability of this formula were presented.

Notice that formula (4) with *r*_0_=0 (i.e., the whole capillary is filled with blood plasma) transforms into the Poiseuille formula:

(6) $$\begin{array}{c} R=\frac{8L}{\pi }{\left(\frac{r_{\text{c}}^{4}}{\mu_{1}}\right)}^{-1}. \end{array}$$

It should be noted that the model described in this section is a rough approximation of RBC motion in a capillary. Obviously, plasma recirculation between cells should affect the flow properties.  Section 3 considers an extended model, assuming the presence of gaps between RBCs, which means accounting for tube hematocrit.

## 3. Discrete Model of Red Cell Transport

**Figure 2:**
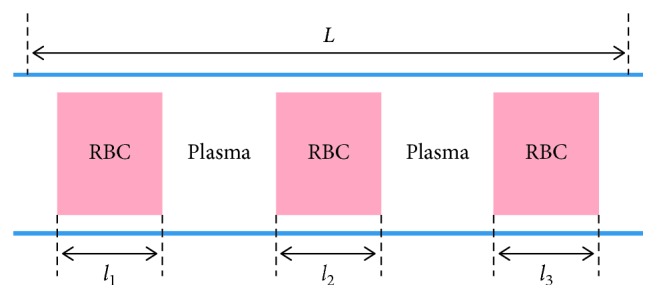
Schematic representation of a discrete stream of erythrocytes in a capillary (the axial section is shown). The RBCs are separated by blood plasma. There is also a plasma lubrication layer between the RBCs and vessel wall.

In [11], RBCs in capillaries are considered as separate rigid bodies immersed in blood plasma, as it is sketched in Figure 2. The RBCs are schematically shown as cylinder-shaped objects (their projections onto the axial section are depicted).

The following formula for the effective resistance *R*^e^ of a capillary, assuming that RBCs move in single-file, is proposed in [11]:

(7) $$\begin{array}{c} R^{\text{e}}=R\left(1+H\beta \right). \end{array}$$

where $H=\hat{L}/L$ is the value of tube hematocrit, and $\beta =\left(\hat{\varrho }/\varrho \right)-1$, where $\hat{L}=\sum_{i}l_{i}$ is the part occupied by RBCs, $\hat{\varrho }$ is the specific resistance for the parts occupied by erythrocytes, and *ϱ* is the specific resistance for the parts filled with blood plasma. Moreover, *R* is the Poiseuille resistance of the vessel filled only with blood plasma, i.e., *R*=*Lϱ*. Obviously, *R*^e^=*R* if *H*=0, and $R^{\text{e}}=\hat{R}\;≔\;\hat{\varrho }L$ if *H*=1. Note that the Obrist et al. [11] neglected the plasma layer between RBCs and the capillary wall, which yields some error. For example, the values 0.1, 0.4, and 1 of *H* correspond to the values 0.082, 0.33, and 0.82 of exact tube hematocrit. Nevertheless, the definition of [11] will be kept for consistency. Note that single-file flow of RBCs is typical for small vessel diameters (down to 10 *μ*m [15]), which holds for the capillary network of the brain.

Formula (7) can be transformed into the following one:

(8) $$\begin{array}{c} R^{\text{e}}=\hat{L}\hat{\varrho }+\left(L-\hat{L}\right)\varrho , \end{array}$$

which means that *R*^e^ is the resistance of serially connected parts corresponding to the intervals *l*_*i*_ and gaps between them.

Note that paper [11] does not propose a precise definition of *β* in (7). In contrast, we are able to compute *β* directly using the expressions (4), (5), and (6); that is, $\hat{\varrho }$ and *ϱ* will be set as follows:

(9) $$\begin{array}{c} \hat{\varrho }=\frac{8}{\pi }{\left(\frac{r_{\text{c}}^{4}-r_{0}^{4}}{\mu_{1}}+\frac{r_{0}^{4}}{\mu_{2}}\right)}^{-1},\\ \varrho =\frac{8}{\pi }{\left(\frac{r_{\text{c}}^{4}}{\mu_{1}}\right)}^{-1},\\ r_{0}=0.3\;\mu \text{m}+0.8r_{\text{c}}. \end{array}$$

## 4. Finite Element Model of Red Cell Transport

**Figure 3:**
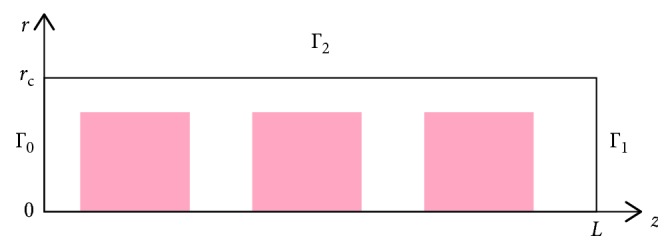
Schematic drawing of the FE domain in cylindrical coordinates; only three RBCs are shown. The RBC subdomain is shown in pink, and the plasma subdomain is colorless.

Similar to the modeling method described in Section 2, the RBCs and blood plasma are considered as one flow with two different viscosities. As in Section 2, the viscosity of blood plasma is assumed to be *μ*_1_=0.001 Pa · s, whereas the viscosity of RBCs is set to be *μ*_2_=0.1 Pa · s to make RBCs effectively rigid. Moreover, it is assumed that the flow is steady state, without transition effects. Therefore, the model is described by the steady state Stokes equation with spatially variable viscosity. As usually, Euler's reference system is used; that is, the flow velocity is computed at each spatial point of the unmovable simulation domain. Assuming that the flow is axisymmetric and all variables depend only on the radial and longitudinal coordinates *r* and *z*, the problem is reduced to a two-dimensional one (Figure 3). Using the linear size and volume of erythrocytes reported in [16, 17], the average length of erythrocytes was estimated to be 3 *μ*m. The FE method is implemented with triangle linear finite elements. The simulation domain is partitioned into 50 × 1000 rectangles in the radial and longitudinal directions, respectively, and each rectangle was divided into two triangles.

Let *u*_*r*_ and *u*_*z*_ be radial and longitudinal flow velocities, respectively, *p* the pressure, and *Ω*=(0, *r*_c_) × (0, *L*).

The model is mathematically formulated in [18] in a weak form, which allows for using spatially discontinuous viscosity functions. With *x*_1_=*r*, *x*_2_=*z*, *u*_1_=*u*_*r*_, *u*_2_=*u*_*z*_, *u*=(*u*_1_, *u*_2_)^*T*^, *p*(*r*, 0)=*p*_0_, and *p*(*r*, *L*)=0, the weak formulation reads in cylindrical coordinates as follows:

(10) $$\begin{array}{c} \int_{\Omega }x_{1}\left(2\mu \left(x_{1},x_{2}\right)\overset{2}{\underset{i,j=1}{\sum }}D_{ij}\left(u\right)D_{ij}\left(v\right)+\frac{u_{1}v_{1}}{x_{1}^{2}}\right)dx-\int_{\Omega }x_{1}p\text{ div}\left(v\right)dx=\int_{\Gamma_{0}}x_{1}p_{0}v_{2}\;dx, \end{array}$$

(11) $$\begin{array}{c} \varepsilon \int_{\Omega }x_{1}pq\;dx-\int_{\Omega }x_{1}\text{ div}\left(u\right)q\;dx=0,\\ {\left.u\right|}_{\Gamma_{2}}=0,\\ {\left.v\right|}_{\Gamma_{2}}=0, \end{array}$$

where *D*_*ij*_(*u*)=1/2((∂*u*_*i*_/∂*x*_*j*_)+(∂*u*_*j*_/∂*x*_*i*_)) and div(*u*)=(*u*_1_/*x*_1_)+(∂*u*_1_/∂*x*_1_)+(∂*u*_2_/∂*x*_2_).

Functions *v*=(*v*_1_, *v*_2_)^*T*^ and *q* are the test ones. The viscosity distribution *μ*(*x*_1_, *x*_2_) is the following discontinuous function:

(12) $$\begin{array}{c} \mu \left(x_{1},x_{2}\right)=\left\{\begin{array}{cc} \mu_{1}=0.001\text{ Pa}\cdot \text{s}, & \mathrm{if}\left(x_{1},x_{2}\right)\in \text{plasma part},\\ \mu_{2}=0.1\text{ Pa}\cdot \text{s}, & \mathrm{if}\left(x_{1},x_{2}\right)\in \text{RBCs part}. \end{array}\right. \end{array}$$

Thus, the RBCs are modeled as fluid parts with a high viscosity to make them effectively rigid.

The model (10)-(11) is equivalent to the following one:

(13) $$\begin{array}{c} \int_{\Omega }x_{1}\left(2\mu \left(x_{1},x_{2}\right)\overset{2}{\underset{i,j=1}{\sum }}D_{ij}\left(u\right)D_{ij}\left(v\right)+\frac{u_{1}v_{1}}{x_{1}^{2}}+\nabla pv\right)dx=0,\\ \varepsilon \int_{\Omega }x_{1}\left(pq-\text{div}\left(u\right)q\right)dx=0, \end{array}$$

(14) $$\begin{array}{c} {\left.p\right|}_{\Gamma_{0}}=p_{0},\\ {\left.p\right|}_{\Gamma_{1}}=0,\\ {\left.u\right|}_{\Gamma_{2}}=0,\\ {\left.v\right|}_{\Gamma_{2}}=0, \end{array}$$

where ∇*p*=(∂*p*/∂*x*_1_, ∂*p*/∂*x*_2_).

**Figure 4:**
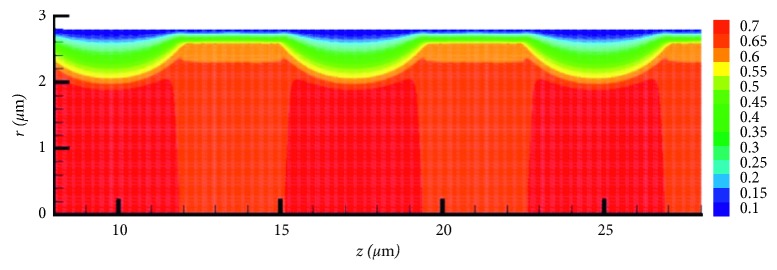
The *z*-component of flow velocity (mm/s). The velocity is smaller in the parts occupied by red cells.

The system (13)-(14) is implemented with finite element method using the FreeFEM++ package [19].  Figure 4 shows the *z*-component of the velocity. The simulation is done for *r*_c_=2.8 *μ*m, *L*=60 *μ*m, *p*_0_=0.63 mmHg, and *H*=0.4. Only a part (20 *μ*m) is presented in Figure 4 for better illustration.

**Figure 5:**
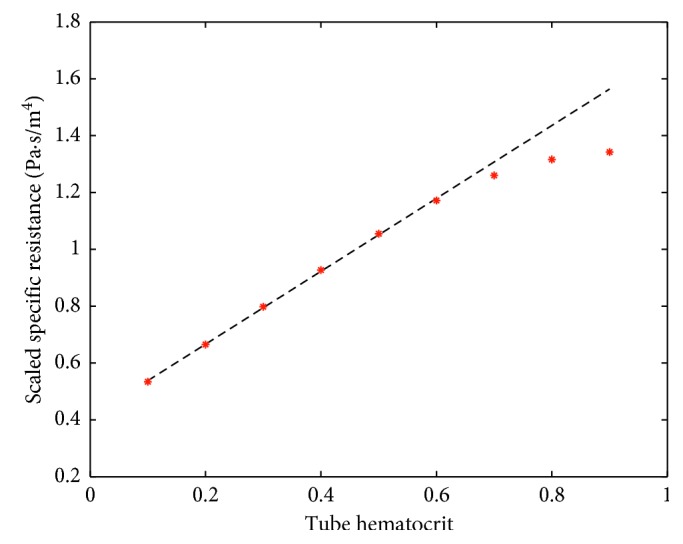
The dependence of specific resistance (red dots) on the value of tube hematocrit for *r*_c_=2.8 *μ*m, computed with the FM model. The dashed line is a good linear approximation of this dependence for *H* ≤ 0.6. The scale factor for the vertical axis is equal to 10^−20^.

Figure 5 shows the dependence of specific resistance on the value of tube hematocrit for *r*_c_=2.8 *μ*m. An almost linear behavior holds in the range *H* ≤ 0.6. The dashed line, 1.28*H*+0.41, is a good linear approximation for this range, which includes practically all realistic values of tube hematocrit.

## 5. Adjustment of the Formula for Capillary Flow Resistance

**Figure 6:**
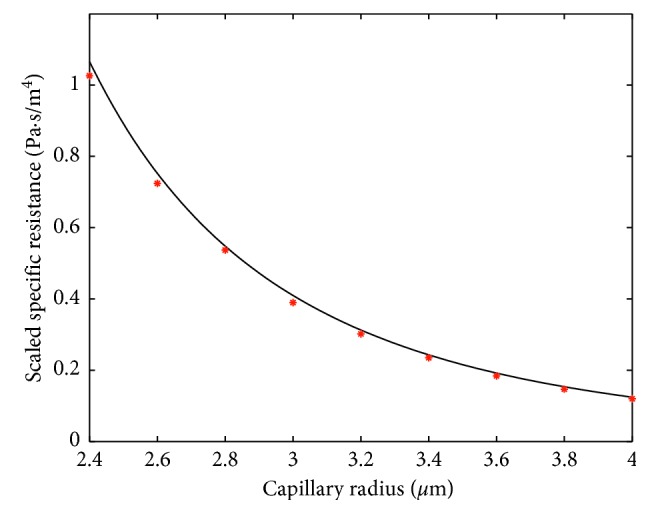
Comparison between analytically (solid line) and FEM-computed (dots) specific resistances in the case of *H*=0.1. The scale factor for the vertical axis is equal to 10^−20^.

**Figure 7:**
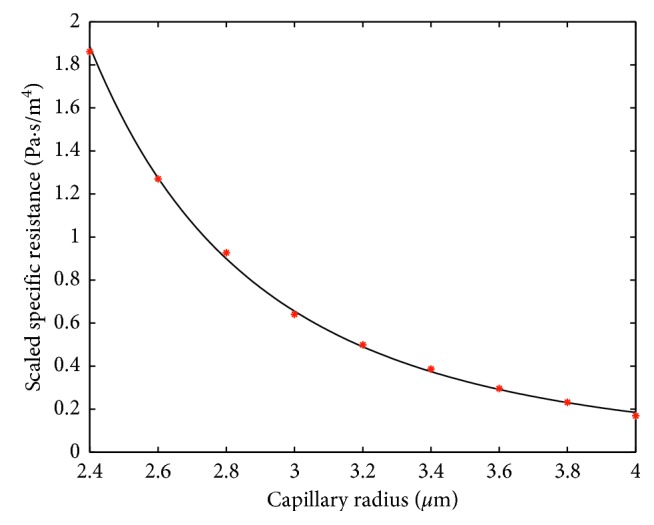
Comparison between analytically (solid line) and FEM-computed (dots) specific resistances in the case of *H*=0.4. The scale factor for the vertical axis is equal to 10^−20^.

Fitting formula (7), or equally (8), to the numerical results is performed by increasing the blood plasma viscosity in (9) to the value *μ*_1_=0.0011 Pa · s for *H*=0.1 and to the value *μ*_1_=0.0012 Pa · s for *H*=0.4. In this case, a very good agreement of this formula with numerical results is shown in Figures 6 and 7. Thus, for fitting the formula (7) to the results of the numerical simulation, we need to increase the viscosity of the plasma when the hematocrit is increased. Notice that the interval from 0.1 to 0.4 covers a large part of the normal tube hematocrit range [16]. Thus, we can interpolate the above values of *μ*_1_ to obtain *μ*_1_(*H*)=10^−3^(16+5*H*)/15. Obviously, *μ*_1_(0.1)=0.0011 and *μ*_1_(0.4)=0.0012.

**Figure 8:**
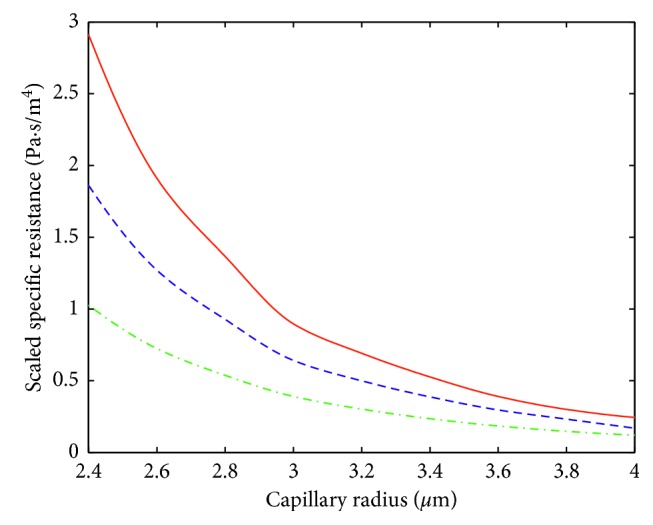
Specific resistances computed with FEM for different values of the hematocrit: *H*=1 (solid red), *H*=0.4 (dashed blue), and *H*=0.1 (dashed-dot green). The scale factor for the vertical axis is equal to 10^−20^.

The influence of hematocrit on the specific resistance is shown in Figure 8. Here, the specific resistances computed by the Finite Element method for different values of hematocrit (*H*=1, *H*=0.4, and *H*=0.1) are presented. The value *H*=1 corresponds to the model used in [7], where the gapless flow of RBCs was assumed. For the tube hematocrit values of 0.1 and 0.4, the corresponding specific resistances approximately differ by factor 2, which points out to the importance of incorporating tube hematocrit in models of capillary blood flow.

Finally, the effective specific resistance is given by the following formula:

(15) $$\begin{array}{c} r^{\text{e}}=\rho +\left(\hat{\rho }-\rho \right)H, \end{array}$$

where *ρ* and $\hat{\rho }$ are defined by (9) with *μ*_1_=*μ*_1_(*H*)≔10^−3^(16+5*H*)/15.

**Figure 9:**
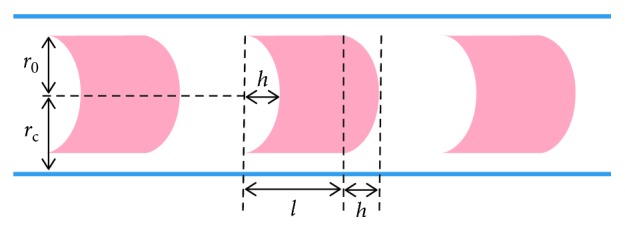
Noncylindrical (deformed) RBCs.

**Figure 10:**
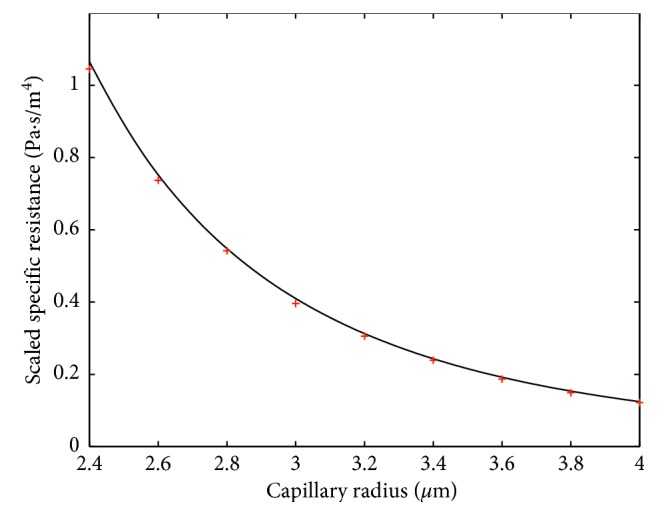
Comparison between analytically (solid line) and FEM-computed (dots) specific resistances for noncylindrical (deformed) RBCs in the case *H*=0.1. The scale factor for the vertical axis is equal to 10^−20^.

**Figure 11:**
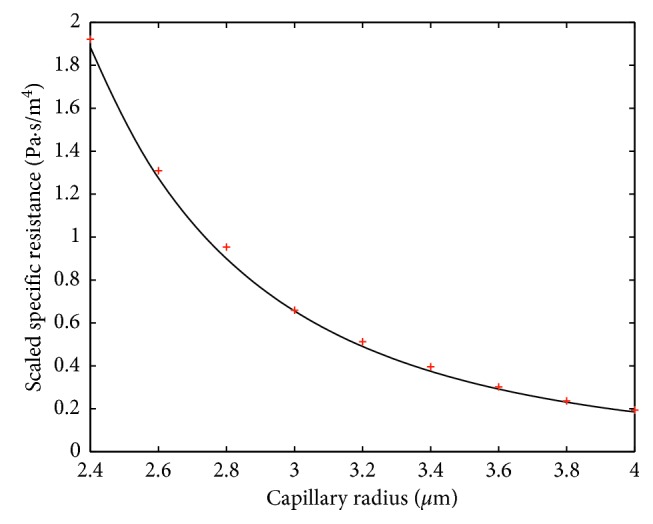
Comparison between analytically (solid line) and FEM-computed (dots) specific resistances for noncylindrical (deformed) RBCs in the case *H*=0.4. The scale factor for the vertical axis is equal to 10^−20^.

Simulation results presented in Figures 6 and 7 correspond to cylinder-shaped erythrocytes with the radius *r*_0_ and length *l*_*i*_=3 *μ*m. To check the robustness of the model with respect to the change of RBC shape, simulations with a modified RBC shape were performed. The new shape, with parabolic front and back parts, is shown in Figure 9. In the simulations, *l*=3 *μ*m and *h*=*l*/3 were used. Figures 10 and 11 show the specific resistance versus capillary radius for the hematocrit values *H*=0.1 and *H*=0.4, respectively. The results were computed both by the FE method and analytic formula (15) with *μ*_1_=*μ*_1_(*H*)≔10^−3^(16+5*H*)/15. A good agreement between the results delivered by the two methods is observed.

## 6. Verification of the Method on Large Capillary Networks

To check the consistency of formula (15), the total resistance of the brain capillary network is computed. Similar to [7], it is assumed that capillaries of the brain are connected in a network having a variable random topology, i.e., a random number of incident edges are generated for each node. The topology is characterized by the average number of incident edges for each node so that 2-edge, 3-edge, 4-edge, etc. random topologies can be considered. Let us explain the network generation in the case of 4-edge topology. First, the set of all nodes, (*i*, *j*, *k*), is generated. For a node (*i*, *j*, *k*), the successive closest nodes, e.g., (*i*+1, *j*, *k*), (*i*, *j*+1, *k*), and (*i*, *j*, *k*+1), are considered, and two of them are randomly chosen. These two nodes are to be connected to (*i*, *j*, *k*) with the corresponding edges (capillaries). The probability that the current node (*i*, *j*, *k*) already has two incident edges, constructed when (*i*, *j*, *k*) was considered as a successive node with respect to the nodes (*i* − 1, *j*, *k*), (*i*, *j* − 1, *k*), and (*i*, *j*, *k* − 1), is close to 1. Therefore, the network constructed in such a way has in average 4-edge topology.

Additionally, it is supposed that the length and radius of capillaries are random values distributed according to data reported in [20]. Moreover, a tube hematocrit level is assigned to all capillaries, and formula (15) is applied to compute the resistance of each capillary. Similar to [7], it is supposed that the network contains blood sources and sinks (inlets and outlets) distributed over the network. They are associated with the arteriolar and venular endpoints, respectively. The calculation of the total resistance is performed by the direct computation of the total blood flux through the capillary network by analogy with electric circuits, i.e., using Kirchhoff's law and solving a large sparse system of linear algebraic equations.

The results yielded by the above sketched procedure are compared with data obtained from a model by Piechnik et al. ([21]) fitted to experimental data of papers [22–24], where intravascular pressures have been measured on animals. Since the model from [21] assumes the parallel connection of all capillaries, which reduces the global hydraulic resistance, the length of capillaries has apparently been increased by factor 10 to be equal 600 *μ*m there. This allowed the authors of [21] to fit the modeled pressure drop in the capillary compartment to the data reported in [22–24]. However, experimentally retrieved parameters of capillaries reported in [20] are the following: the mean length is equal to 57.4 *μ*m and mean diameter equals 5.9 *μ*m (M1-mosaic). We use these realistic values and, additionally, different levels of tube hematocrit in the test runs.

**Figure 12:**
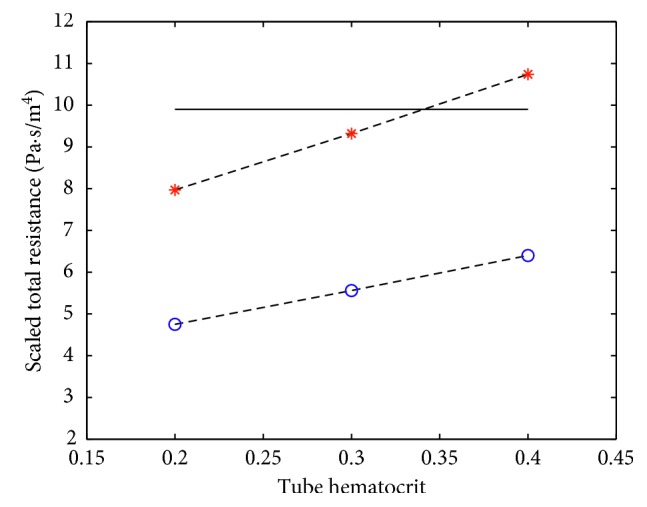
The scaled total resistance of the brain capillary network for different values of tube hematocrit: solid line—Piechnik's model immediately based on experimental data; red asterisks—the case of 3-edge random topology; blue hollow circles—the case of 4-edge random topology. The dashed lines demonstrate linear dependency of the resistance on the hematocrit level. The scale factor for the vertical axis is equal to 10^−7^. Note that this simulation shows the sensitivity of the method against the change of the hematocrit level and random topology.

The computation, using different random topologies, of the hydraulic resistance of the entire adult cerebral capillary network containing 756 million capillaries is presented in Figure 12. Here, the horizontal solid black line corresponds to the value of 9.9 · 10^7^(Pa · s/m^3^) computed with the Piechnik et al. model ([21]) based on experimental data. The red stars show values of the total brain resistance calculated by the authors' method assuming 3-edge random topology and different values of tube hematocrit. The dashed line connecting the red stars demonstrates a linear dependence of the result on the hematocrit level. The blue hollow circle dots stand for 4-edge random topology. It is seen that the case of 3-edge random topology (along with the hematocrit level of 0.35) yields the best approximation of the value delivered by Piechnik's model that, generally speaking, interpolates experimental data. Therefore, it is established that 3-edge random topology should be more consistent with the structure of the brain capillary network, which is in agreement with physiological data. Note that 4-edge (not quite realistic) random topology was found in [7] to be the best one because of ignoring the tube hematocrit level (it was always equal to 1 by default).

## 7. Computing the Pressure Distribution in the Germinal Matrix Accounting for Tube Hematocrit

As it is seen from Figure 8, the hydraulic resistance of a capillary essentially depends on the level of tube hematocrit. Thus, the pressure distribution in a cerebral capillary network should be significantly affected by the hematocrit level. This is especially important in the case of the subependymal germinal matrix, which is a specific part of the immature brain with high vascularity and a fragile capillary network [25], because most hemorrhages originate from this structure [26].

To calculate the pressure distribution in the germinal matrix, accounting for the hematocrit level, the approach proposed in [7] is used in the current paper with some modifications. Recall first that the total vascular system of the infant brain was described in [7] using a model proposed in [21]. This model comprises 19 levels of brain vessels: 9 levels of arterioles, 9 levels of venules, and 1 level of capillaries. All vessels at each level *i*, *i*=1,…, 19, are connected in parallel. In [7], to adapt the model to infants, the numbers of vessels, their lengths, and their diameters were reduced by dividing the original values over 12 − 1.22|*i* − 10|, 1+0.14|*i* − 10|, and 1+0.11|*i* − 10|, respectively, which fits the CBF to a typical value of the infants brain corresponding to the age of 25 weeks [27, 28]. Resistances of noncapillary vessels are given by Poiseuille's formula (6) with the apparent viscosity set to be equal to 0.003 Pa · s ([21]). The capillary level is assumed to consist of two networks corresponding to the germinal matrix and the rest part of the brain, respectively.

**Figure 13:**
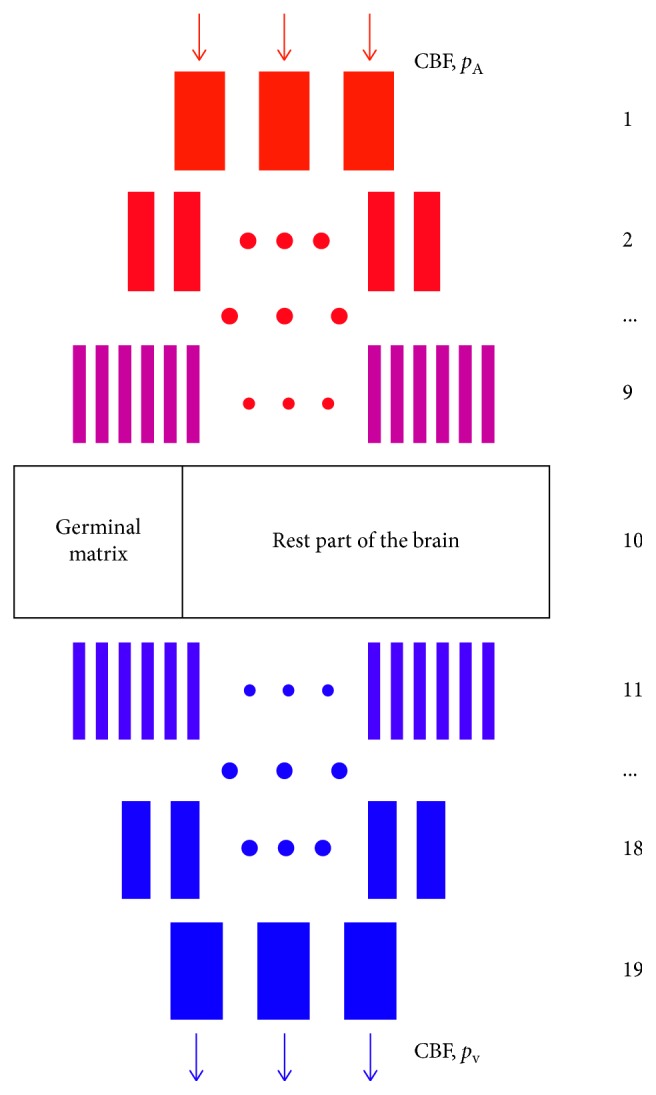
A vascular brain model used in [7] for the calculation of the pressure drop in the germinal matrix. The germinal matrix and the rest part of brain are considered as lumped objects. The model is a modification of that developed in [21].

In the current paper, it is supposed that these networks have 3-edge random topology, and the capillary mean length and diameter are equal to 57.4 *μ*m and 5.9 *μ*m, respectively ([20]). The total resistances *R*_GM_ (of the germinal matrix) and *R*_B_ (of the rest part of the brain) are computed as it is outlined in Section 6, assuming that formula (15) is used for computing capillary resistances. To compute the pressure drop in the germinal matrix, the capillary level is replaced with two parallel connected lumped objects having the resistances *R*_GM_ and *R*_B_, which yields the total resistance of the whole 10th level (Figure 13):

(16) $$\begin{array}{c} R_{10}={\left(R_{\text{GM}}^{-1}+R_{\text{B}}^{-1}\right)}^{-1}. \end{array}$$

For the other levels, the resistances are calculated as

(17) $$\begin{array}{c} R_{i}=\frac{{\bar{R}}_{i}}{N_{i}},\;i=\bar{1,19},\;i\neq 10, \end{array}$$

where ${\bar{R}}_{i}$ is the resistance of a single vessel of *i* th level and *N*_*i*_ the number of vessels of *i* th level ([21]). The total resistance is given as

(18) $$\begin{array}{c} R_{\text{T}}=\overset{19}{\underset{i=1}{\sum }}R_{i}, \end{array}$$

and the total flow is given by the following formula:

(19) $$\begin{array}{c} Q=\frac{\left(p_{\text{A}}-p_{\text{V}}\right)}{R_{\text{T}}}, \end{array}$$

where *p*_A_ and *p*_V_ are the arterial and venous (intracranial) pressures for infants. Finally, the pressure drops in the 10th level of the model, and therefore, in the germinal matrix, it is given as

(20) $$\begin{array}{c} \Delta p=QR_{10}. \end{array}$$

Remember that Δ*p* is the difference of pressures exerted on the blood sources and sinks (inlets and outlets) distributed in the germinal matrix, and, therefore, Δ*p* is the driving force of flow.

Parameters of a brain corresponding to infants of 25 weeks' gestational age (Table 1) are used.

**Figure 14:**
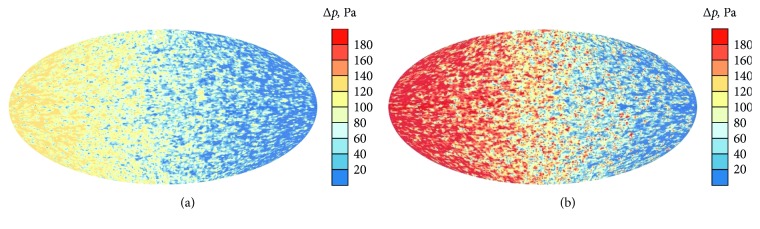
Pressure drop distributions in the longitudinal cross-section of the germinal matrix: (a)  *H*=0.1; (b)  *H*=0.4.

Two tube hematocrit values, 0.1 and 0.4, that are close to extreme levels [16] are taken. In both cases, the resistances of the germinal matrix and the rest part of the brain, the pressure drop in the germinal matrix, and the cerebral blood flow are presented in Table 2. It is seen that the pressure drop in the germinal matrix varies by factor 1.6 if the hematocrit level increases from 0.1 to 0.4. The corresponding pressure drop distributions in a longitudinal cross-section of the germinal matrix are shown in Figure 14. The elliptic shape is chosen for better visualization. The gradient is caused by a heterogeneous distribution of inlets and outlets in the germinal matrix (see the middle of Section 4 of [7]).

An essential difference in the pressure drop distributions is clearly seen there.

## 8. Conclusion

When creating a model of cerebral capillary network, it is necessary to randomly assign diameter and length to almost billion of capillaries and to compute the RBC flow resistance for each capillary. Therefore, it is important to have a simple analytical formula for the resistance accounting for the hematocrit level. In the current paper, such a formula is proposed and numerically verified through finite element simulations. Accounting for hematocrit will justify and enhance models of cerebral capillary networks, allowing us to study the danger of vessel rupture in the germinal matrix, dependence on the hematocrit level.

Bearing in mind that the main goal of our study is simulation of large capillary networks, we neglect some fine effects. For example, we assume that the hematocrit level is constant over the capillary network, the plasma lubrication layer between the erythrocyte flow and the capillary wall depends on the capillary diameter only, the effect of bifurcation at network nodes is dropped because of approximate homogeneity of capillaries, and the effect of ESL (endothelial surface layer) is not accounted for. We believe that the effect of such simplifications is not damaging in context of modeling large capillary networks. Our future intentions include enhancements of the current model, in particular through including the bifurcation and ESL effects. We are planning the investigation of fluid interaction with ESL using homogenization theory for partial differential equations.

## Figures and Tables

**Table 1:** Average data corresponding to the age of 25 gestational weeks.

Parameter	Name	Value	Source
*N*_A_	Number of capillaries of the adult brain	756 · 10^6^	[21]
*w*_A_	Weight of the adult brain	1.2 kg	[21]
*w*	Weight of the infant brain of 25 gestational weeks	0.1 kg	[29]
*p*_GM_	Weight part of the germinal matrix	0.023	[30]
*w*_GM_	Weight of the germinal matrix for infants of 25 gestational weeks, *w*_GM_≔*p*_GM_*w*	2.3 g	—
*κ*	Capillary density correction factor for the germinal matrix	1.5	[31]
*N*_GM_	Number of capillaries of the germinal matrix for infants of 25 gestational weeks, *N*_GM_≔*κw*_GM_*N*_A_/*w*_A_	2.17 · 10^6^	—
*N*_B_	Number of capillaries of the rest part of the brain of 25 gestational weeks, *N*_B_≔(*w* − *w*_GM_)*N*_A_/*w*_A_	61.55 · 10^6^	—
*p*_A_/*p*_V_	Arterial/venous pressure	34/5 mmHg	[28, 32]

**Table 2:** Dependence of computed network characteristics on the tube hematocrit level.

Characteristic	*H*=0.1	*H*=0.4
Resistance of the germinal matrix, *R*_GM_(Pa · s/m^3^)	150 · 10^8^	229 · 10^8^
Resistance of the rest part of the brain, *R*_B_(Pa · s/m^3^)	8.2 · 10^8^	13.2 · 10^8^
Pressure drop in the germinal matrix, Δ*p* (Pa)	129.2	203.3
CBF (ml/min)	9.97	9.77

## Data Availability

The data used can be found in the references cited in this paper.

## References

[1] Secomb T. W., Hsu R., Pries A. R. (2001). Motion of red blood cells in a capillary with an endothelial surface layer: effect of flow velocity. *American Journal of Physiology-Heart and Circulatory Physiology*.

[2] Fedosov D. A., Caswell B., Popel A. S., Karniadakis G. E. (2010). Blood flow and cell-free layer in microvessels. *Microcirculation*.

[3] Sarelius I. H. (1989). Microcirculation in striated muscle after acute reduction in systemic hematocrit. *Respiration Physiology*.

[4] Hudetz A. G., Wood J. D., Biswal B. B., Krolo I., Kampine J. P. (1999). Effect of hemodilution on RBC velocity, supply rate, and hematocrit in the cerebral capillary network. *Journal of Applied Physiology*.

[5] Reinhart W. H., Piety N. Z., Shevkoplyas S. S. (2017). Influence of feeding hematocrit and perfusion pressure on hematocrit reduction (Fåhraeus effect) in an artificial microvascular network. *Microcirc*.

[6] Hudetz A. G. (1990). Dependence of cerebral capillary hematocrit on red cell flow separation at bifurcations: a computer simulation study. *Advances in Experimental Medicine and Biology*.

[7] Botkin N. D., Kovtanyuk A. E., Turova V. L., Sidorenko I. N., Lampe R. (2018). Direct modeling of blood flow through the vascular network of the germinal matrix. *Computers in Biology and Medicine*.

[8] Evans E. A., Skalak R. (1980). *Mechanics and Thermodynamics of Biomembranes*.

[9] Secomb T. W., Skalak R., Özkaya N., Gross J. F. (1986). Flow of axisymmetric red blood cells in narrow capillaries. *Journal of Fluid Mechanics*.

[10] Chebbi R. (2015). Dynamics of blood flow: modeling of the Fåhræus-Lindqvist effect. *Journal of Biological Physics*.

[11] Obrist D., Weber B., Buck A., Jenny P. (2010). Red blood cell distribution in simplified capillary networks. *Philosophical Transactions of the Royal Society A: Mathematical, Physical and Engineering Sciences*.

[12] Lücker A., Weber B., Jenny P. (2015). A dynamic model of oxygen transport from capillaries to tissue with moving red blood cells. *American Journal of Physiology-Heart and Circulatory Physiology*.

[13] Starovoitov V. N. (2003). Behavior of a rigid body in an incompressible viscous fluid near a boundary. *Free Boundary Problems*.

[14] Secomb T. W. (2017). Blood flow in the microcirculation. *Annual Review of Fluid Mechanics*.

[15] Takeishi N., Imai Y. (2017). Capture of microparticles by bolus flow of red blood cells in capillaries. *Scientific Reports*.

[16] Wang T., Xing Z. (2010). Characterization of blood flow in capillaries by numerical simulation. *Journal of Modern Physics*.

[17] Stadler A., Linderkamp O. (1989). Flow behavior of neonatal and adult erythrocytes in narrow capillaries. *Microvascular Research*.

[18] Tabata M. (1996). Finite element analysis of axisymmetric flow problems and its application. *Journal of Applied Mathematics and Mechanics*.

[19] Hecht F. (2012). New development in freefem++. *Journal of Numerical Mathematics*.

[20] Cassot F., Lauwers F., Fouard C., Prohaska S., Lauwers-Cances V. (2006). A novel three-dimensional computer-assisted method for a quantitative study of microvascular networks of the human cerebral cortex. *Microcirculation*.

[21] Piechnik S. K., Chiarelli P. A., Jezzard P. (2008). Modelling vascular reactivity to investigate the basis of the relationship between cerebral blood volume and flow under CO_2_ manipulation. *NeuroImage*.

[22] Bevan J. A., Halpern W., Mulvany M. J. (1991). *The Resistance Vasculature*.

[23] Gore R. W. (1974). Pressures in cat mesenteric arterioles and capillaries during changes in systemic arterial blood pressure. *Circulation Research*.

[24] Zweifach B. W., Lipowsky H. H. (1977). Quantitative studies of microcirculatory structure and function. III. Microvascular hemodynamics of cat mesentery and rabbit omentum. *Circulation Research*.

[25] Volpe J. J. (2008). *Neurology of the Newborn*.

[26] Hambleton G., Wigglesworth J. S. (1976). Origin of intraventricular haemorrhage in the preterm infant. *Archives of Disease in Childhood*.

[27] Lampe R., Botkin N., Turova V., Blumenstein T., Alves-Pinto A. (2014). Mathematical modelling of cerebral blood circulation and cerebral autoregulation: towards preventing intracranial hemorrhages in preterm newborns. *Computational and Mathematical Methods in Medicine*.

[28] Sidorenko I., Turova V., Botkin N. (2018). Modeling cerebral blood flow dependence on carbon dioxide and mean arterial blood pressure in the immature brain with accounting for the germinal matrix. *Frontiers in Neurology*.

[29] Archie J. G., Collins J. S., Lebel R. R. (2006). Quantitative standards for fetal and neonatal autopsy. *American Journal of Clinical Pathology*.

[30] Kinoshita Y., Okudera T., Tsuru E., Yokota A. (2001). Volumetric analysis of the germinal matrix and lateral ventricles performed using MR images of postmortem fetuses. *American Journal of Neuroradiology*.

[31] Ballabh P., Braun A., Nedergaard M. (2004). Anatomic analysis of blood vessels in germinal matrix, cerebral cortex, and white matter in developing infants. *Pediatric Research*.

[32] Skinner J. R., Milligan D. W., Hunter S., Hey E. N. (1992). Central venous pressure in the ventilated neonate. *Archives of Disease in Childhood*.

